# How artificial light at night may rewire ecological networks: concepts and models

**DOI:** 10.1098/rstb.2022.0368

**Published:** 2023-12-18

**Authors:** Dirk Sanders, Myriam R. Hirt, Ulrich Brose, Darren M. Evans, Kevin J. Gaston, Benoit Gauzens, Remo Ryser

**Affiliations:** ^1^ Environment and Sustainability Institute, University of Exeter, Penryn, Cornwall TR10 9FE, UK; ^2^ German Centre for Integrative Biodiversity Research (iDiv) Halle-Jena-Leipzig, 04103 Leipzig, Germany; ^3^ Institute of Biodiversity, Friedrich Schiller University Jena, 07737 Jena, Germany; ^4^ School of Natural and Environmental Sciences, Newcastle University, Newcastle upon Tyne NE1 7RU, UK

**Keywords:** light pollution, ecological communities, human impact, niche, activity patterns

## Abstract

Artificial light at night (ALAN) is eroding natural light cycles and thereby changing species distributions and activity patterns. Yet little is known about how ecological interaction networks respond to this global change driver. Here, we assess the scientific basis of the current understanding of community-wide ALAN impacts. Based on current knowledge, we conceptualize and review four major pathways by which ALAN may affect ecological interaction networks by (i) impacting primary production, (ii) acting as an environmental filter affecting species survival, (iii) driving the movement and distribution of species, and (iv) changing functional roles and niches by affecting activity patterns. Using an allometric–trophic network model, we then test how a shift in temporal activity patterns for diurnal, nocturnal and crepuscular species impacts food web stability. The results indicate that diel niche shifts can severely impact community persistence by altering the temporal overlap between species, which leads to changes in interaction strengths and rewiring of networks. ALAN can thereby lead to biodiversity loss through the homogenization of temporal niches. This integrative framework aims to advance a predictive understanding of community-level and ecological-network consequences of ALAN and their cascading effects on ecosystem functioning.

This article is part of the theme issue ‘Light pollution in complex ecological systems’.

## 1. Introduction

Artificial light at night (ALAN) from a variety of anthropogenic light sources is spilling into large areas of terrestrial, freshwater and coastal environments around the world, markedly changing natural light regimes [1–4]. This impact, while most intense in urban contexts, extends over a much greater geographical area through rural light installations, traffic and skyglow, where ALAN is scattered far into the landscape through atmospheric water, dust and gas molecules [4,5]. Research into the impact of ALAN has documented the responses of many organisms to this widespread and increasing anthropogenic environmental pressure [6]. Biological responses to ALAN exposure are particularly strong for physiology and behaviour, with different aspects of organismal biology affected either negatively or positively [6–8]. This indicates that by changing the environmental context, ALAN has a strong impact on the performance of a wide range of species. However, while many single-species responses are well documented, a comprehensive understanding is still lacking of how ALAN causes changes to the ways that species interact, and thus how it affects whole ecological communities and the ecological processes that they deliver [9–12].

Interactions between species drive ecosystem functions such as biomass production, top-down control, pollination and community stability. Therefore, to predict the impact of stressors on such functions, we need to study the responses of species interactions embedded within their community. For example, a whole community approach has been crucial in understanding climate change impacts on ecosystems [13–15], particularly with a focus on a shift in interactions [16,17]. These interactions between species can be mapped in food webs or networks, allowing identification of the functional role associated with each species or group of species, and prediction of responses to single extinctions and extinction series [18,19]. The past decade has seen significant advances in the theoretical understanding, construction, analysis and application of complex species interactions networks [20,21]. Ecological networks describe the interactions between species, the underlying structure of communities and the function and stability of ecosystems [22]. They have the potential to quantify the effects of human activities on a wide range of complex ecological interactions [23,24]. ALAN has been shown to change the way in which species interact, with evidence from plant–pollinator networks [12,25–28] and food webs [12,29–32] and with consequences for ecosystem functions [25,31]. Yet, the understanding of ALAN impacts on ecosystem functions and stability is still very limited, primarily due to the complexity of empirical assessments of community- and ecosystem-level responses in nature. Modelling approaches offer the opportunity to deal with such complexity by reducing nature to a few fundamental processes, yet they have virtually never been applied in research on the biological impacts of light pollution. Here, we assess the current understanding of community-wide ALAN impacts and, on this basis, conceptualize and review four major pathways by which ALAN may affect ecological interaction networks. Using a simple allometric–trophic network (ATN) model, we then show how ALAN-induced shifts in temporal niche overlap can affect community persistence.

### (a) Concepts and review

**Figure 1.**
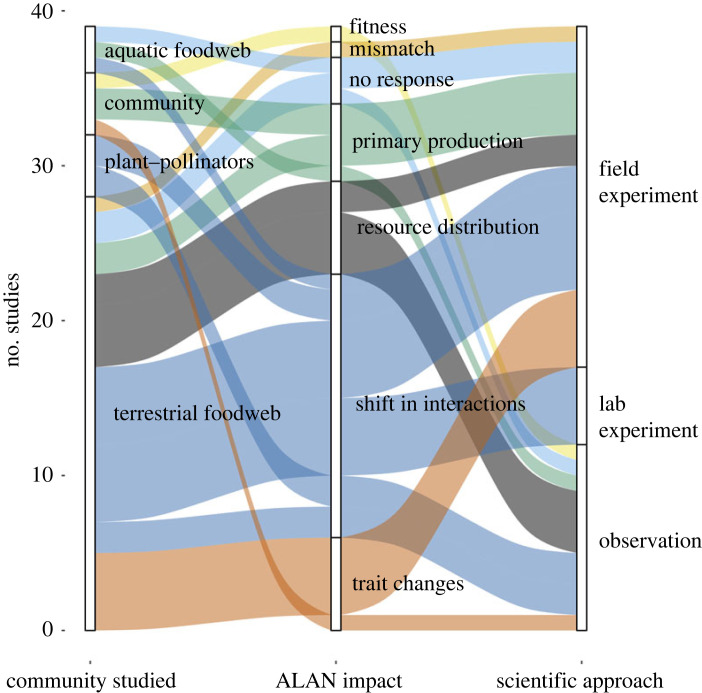
Flow diagram showing the number of studies included in this review that investigate the impact of ALAN on ecological communities. The numbers show the type of community investigated (food web, plant–pollinator community or single trophic-level community, e.g. plants), the main pathways by which the ALAN impact is transmitted in the community (including two studies that report no impact) and the scientific approach used by the authors. The colours in the diagram indicate ALAN impact pathways. (Online version in colour.)

To assess the scientific basis of current understanding of community-wide ALAN impacts, to highlight emerging patterns and to point to gaps in knowledge, we conducted a literature review using Web of Science on 18 May 2023. We searched with the string (‘artificial light at night’ OR ‘light pollution’ OR ‘light at night’ OR ‘anthropogenic light at night) AND (network OR ‘food web’ OR ‘trophic web’). To be included, studies needed to report primary research on ALAN exposure on food webs and networks, or primary producer communities with the potential of cascading effects to higher trophic levels. The search was complemented with literature collected by an expert in the field (K. J. Gaston). We initially scanned 146 manuscripts, with the final number of 39 studies included in this review. All studies were published in the past 10 years, indicating that this is a recently emerging research field. We found studies on plant–pollinator communities, food webs, and plant and microbial communities (figure 1; electronic supplementary material, table S1).

The majority of research used field experiments to manipulate ALAN levels for natural or semi-natural communities (figure 1). This was followed by observations of ALAN-exposed communities and a few laboratory experiments. Some studies combined field and laboratory experiments to gain a better mechanistic understanding.

The main impacts recorded in the studies were on primary production, resource distribution, traits and species interactions. Two studies found no responses, and two single studies reported mismatches between species and direct fitness effects. Considering that shifts in species interactions and traits both describe a change in functional role of species, this appears to be the most common pathway uncovered so far. This pattern of outcomes may well depend on the scientific approaches used. For example, studies investigating environmental filtering at larger spatial scales are urgently needed, but it may be challenging to identify the impact of ALAN in isolation from other impacts. A powerful approach to research ALAN impacts of ecological networks would be the use of a large-scale paired design, with both ALAN environments and natural environments that are not confounded by ALAN sharing cooccurring stressors, such as habitat destruction, heat, pollution or noise.

**Figure 2.**
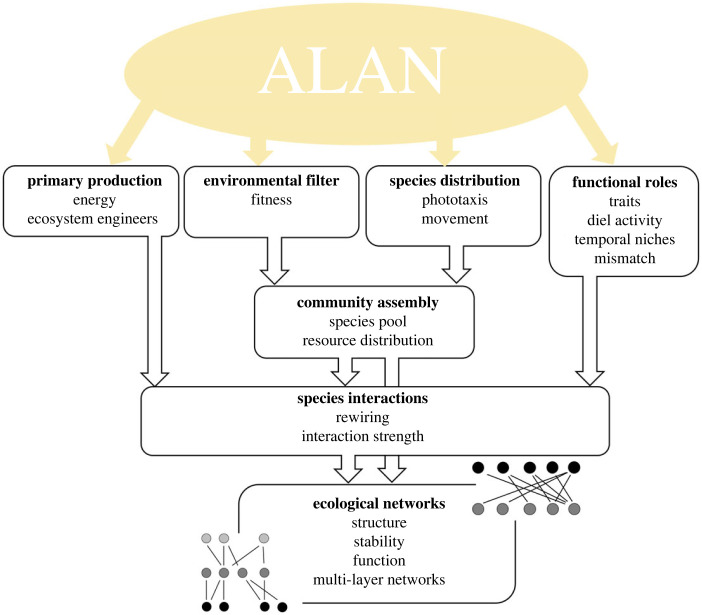
A conceptual framework linking ALAN impact to changes in ecological interaction networks. ALAN impacts ecological-network assembly and species interactions via four different pathways. These have consequences for network structure, stability and the functions provided by the communities. (Online version in colour.)

Based on the studies, we conceptualize and review pathways by which ALAN may affect ecological interaction networks that categorize the variety of community-level responses reported in the literature. We suggest four major pathways: (i) impacting primary production and other plant traits; (ii) acting as an environmental filter on community assembly; (iii) changing species distributions and movements in the landscape with consequences for resource availability; and (iv) changing functional roles and niches by affecting activity patterns and prey/host detectability (figure 2). All these pathways can modify community structure and energy flow, leading to a change in functional properties of these communities.

### (b) Primary production

Studies have found that primary producers respond to ALAN with changes in traits, phenology, biomass and structure [31,33–36]. These changes at the base of food webs can have severe impacts on species interactions and the functioning of whole communities. For instance, changes to flowering phenology can impact plant–pollinator interactions and have fitness consequences for the plants [25]. By modulating primary production, ALAN may also cause bottom-up cascading effects. So far, there is only limited evidence for this, potentially because these effects can be compensated by increased herbivory [31,35]. Moreover, plants are important ecosystem engineers controlling many aspects of the physical environment [37,38]. Therefore, by inducing structural changes, ALAN exposure can have indirect consequences for the strength of food web interactions [39,40].

### (c) Environmental filter

By creating novel environmental conditions, ALAN exposure can impact on the fitness of species, with these new conditions acting as an environmental filter that restructures the local species pool [9,41,42]. This has consequences for the assembly of ecological communities at various spatial scales. For example, ALAN exposure changed the structure of local marine epifaunal communities, with some species being inhibited while others showed a positive response [43]. However, these effects may take longer to become fully apparent than the timeframe available in the majority of current experiments. For instance, the negative impact of ALAN of different spectra on macro-moth communities became evident only after 2 years of exposure [44]. ALAN can restructure local communities especially by excluding species with highly sensitive traits that lead to negative fitness consequences [8]. The results of a meta-analysis suggest that strictly nocturnal species are more likely to suffer, especially when they have very low light level thresholds [6]. By contrast, diurnal species can show positive responses such as increasing foraging activity [6], but these apparently positive effects may have profoundly negative fitness consequences in the long term. There is still very limited understanding of how ALAN as an environmental filter impacts populations, communities and ecosystems at larger scales. It may be difficult to separate the direct impact of ALAN from indirect effects through, for example, the shift in competition owing to the change in light regime [34,45,46]. However, we can expect the removal of sensitive species from communities [25].

### (d) Species distribution

Phototaxis—the attraction and avoidance of species to light sources—has been shown to impact the distribution of mobile species, such as cougars, bats, arthropod predators and various flying insect groups [47–53]. Predatory arthropods, e.g. carabid beetles and spiders, can become more abundant in areas with ALAN exposure, an effect that in one short-term study was only measurable during the night [54]. However, in another experiment conducted over multiple years, ALAN had a long-lasting impact on the distribution throughout the day, thereby demonstrating a systematic impact of ALAN [55]. Many bat species have been shown to exploit the aggregation of flying insects around light sources [53], an effect that may be driven by prey availability rather than direct response to light levels [52]. Positive phototaxis, such as flight-to-light, has been shown to redistribute many species, and this is likely to have important fitness implications [41], either by disrupting important life cycle events and feeding or by causing exhaustion and mortality. Some bat species (e.g. [52,53]) and cougars [51] avoid light-polluted areas for hunting. Further, prey species can become more abundant in lit areas, thereby changing the pattern of resource availability in the landscape. This has a particularly strong effect on the border between freshwater and terrestrial ecosystems [56–60]. Positive and negative phototaxis thereby change the encounter rate between predator and prey and the trophic structure in artificially illuminated areas. Communities that are exposed to ALAN may therefore experience an increased influx of some prey and predators, while other species will be driven away, e.g. nocturnal pollinators. This can lead to the long-term redistribution of species, especially if it happens at larger spatial scales. These dynamics can increase predation/consumption and energy flux, which may then result in larger body sizes [58,60]. Overall, these alterations may also affect the roles of species within their community.

### (e) Functional roles

Diel activity patterns in species and their plasticity have important implications for shifts in functional roles [1,9,61] and have been identified as a crucial trait to predict responses to ALAN exposure [1].

For example, diurnal or crepuscular (dawn- and dusk-active) species can extend their activity into the night when ALAN exposure changes the night-time light levels and unlocks the nocturnal niche for them. This plasticity in behaviour may root in the response of species to naturally changing light conditions, i.e. the moon cycle and seasonal daylength. Activity shifts in response to ALAN have been shown for visual predators [61,62] and also for parasitoids [31], which use mostly chemical cues to find their hosts. By contrast, nocturnal species may retreat from areas that are exposed to ALAN, reducing their activity or their activity range [6,63]. These shifts in activity patterns have important implications for species interactions and food web structure. For instance, many bird species elongate their diel activity by starting earlier and resting later in the day [64,65], which means they may encounter different prey and shift their focus on some prey types, while these birds will also encounter different predators. Miller *et al.* [61] studied how ALAN is changing predation by two ladybird species that hunt during the night, with one of them relying on visual prey cues. They found that only predation by the visual predator was strongly affected by ALAN. Similarly, diurnal parasitoids can use the night-time niche under white ALAN, leading to increased top-down control [31]. However, this effect is disrupted under monochromatic spectra, which impaired their diurnal performance leading to a reduction in host suppression and increased competition between herbivores [46]. ALAN has been shown to switch the night-time behaviour of marine fish from being inactive to predatory behaviour, with consequences for sessile prey assemblages [66]. However, light conditions are not the only factor constraining temporal niches. Colder temperatures at night, for instance, may still lock the nocturnal niche for some species that could exploit the higher light levels; however, we can expect that climate change-driven night warming is likely to increase the shift of many species' activity into the night [61,67]. An in-depth knowledge of nocturnal and diurnal community structures and their functions would help to predict how ALAN impacts these communities. Especially the focus on the nocturnal niche (as a temporal niche for activity), and how that is impacted by different levels and quality of ALAN in the context of naturally complex communities, will provide mechanistic insights. It will allow prediction of the vulnerability of species to these changes, e.g. through predator–prey encounter rates. Trophic niche shifts can also be driven by changes to resource/prey detectability or perceived exposure of prey to potential predators under ALAN [68,69]. Prey behaviour can also change in response to ALAN, making them more vulnerable to predation. For example, moths demonstrate a significant reduction in power-diving as an important antipredator behaviour when exposed to ALAN, and this may add to the accessibility of insects as prey for bats around light sources [70].

Overall, the reorganization of niche spaces by ALAN can lead to a rewiring of food web or network interactions by changing the nocturnal niche and encounter rates between species. Rewiring includes strengthening or weakening existing links and the establishment of new links and removal of old links from the network. For example, a diurnal predator may overexploit resources through higher levels of activity [67] or shift to novel prey species, or a nocturnal prey species may reduce its activity and become unavailable to a predator. Furthermore, if consumers expand their trophic niche (i.e. increase their generalism) in response to ALAN, this can reduce modularity (clustering) in networks and impact community stability.

### (f) Expected consequences for ecological networks

These four pathways can work in concert to change ecological networks. The reorganization of networks under ALAN will be driven by the response of light-sensitive species, leading to shifts in communities and species interactions through movement and changes in activity. Depending on species responses, we can make some tentative predictions that ALAN will cause greater generalism in some diurnal consumer species using the lit nocturnal niche, thereby reducing modularity (the number of subnetworks), increasing connectance (the proportion of realized links in the food web) and potentially reducing food web stability. Reduced stability may be driven by resource overexploitation, increased niche overlap and competition between consumers, and lost temporal refuges for prey. We can further expect extinctions and reduced densities for strictly nocturnal species if ALAN exposure leads to reduced activity and habitat use [25,63]. Any initial changes through these processes will cause a phase transition, new equilibria or even community collapse at the food web/network level through biotic interactions. Therefore, by severely impacting community assembly and changing the way species interact, ALAN exposure may result in the simplification of communities leading to destabilizing dynamics or cascading extinctions with consequences for ecosystem functions. Due to the entangled nature of ecological networks, there may also be feedback effects from one ALAN impact pathway to another. For example, changes in activity patterns or the distribution of consumers can cascade down to primary producers and this could counteract a positive influence of ALAN on plant biomass [35]. The measurable changes to network assembly will depend on the spatial scale investigated and the spatial patterns of ALAN. The same amount of ALAN may have differing effects on communities at the landscape scale, depending on how it is distributed. A patchy ALAN exposure can lead to a similarly patchy distribution of species, but because both lit and unlit areas are available it may allow for some unlit refuges to remain. By contrast, the light exposure in urban areas or through sky glow will impact communities at a larger scale, potentially leading to effects that are not reversible. Species interactions happen at a local scale (as in our model in §2) and this is where we can probably, at least initially, expect the biggest impact.

## 2. Modelling

Interactions between species can only occur when they meet in space and time. ALAN can alter both spatial and temporal niches of organisms. Here, we assess how ALAN-driven shifts in diel activity patterns impact species coexistence. Therefore, we developed a food web model simulating the dynamics of trophically interacting species. We used this model to manipulate how ALAN is changing encounter rates between diurnal, crepuscular and nocturnal species based on different scenarios. We then compared species persistence in the scenarios to a null model without ALAN-driven changes. The detailed description of the model used can be found in the electronic supplementary material. The mode was run using the ATNr package [71].

### (a) Null model

**Figure 3.**
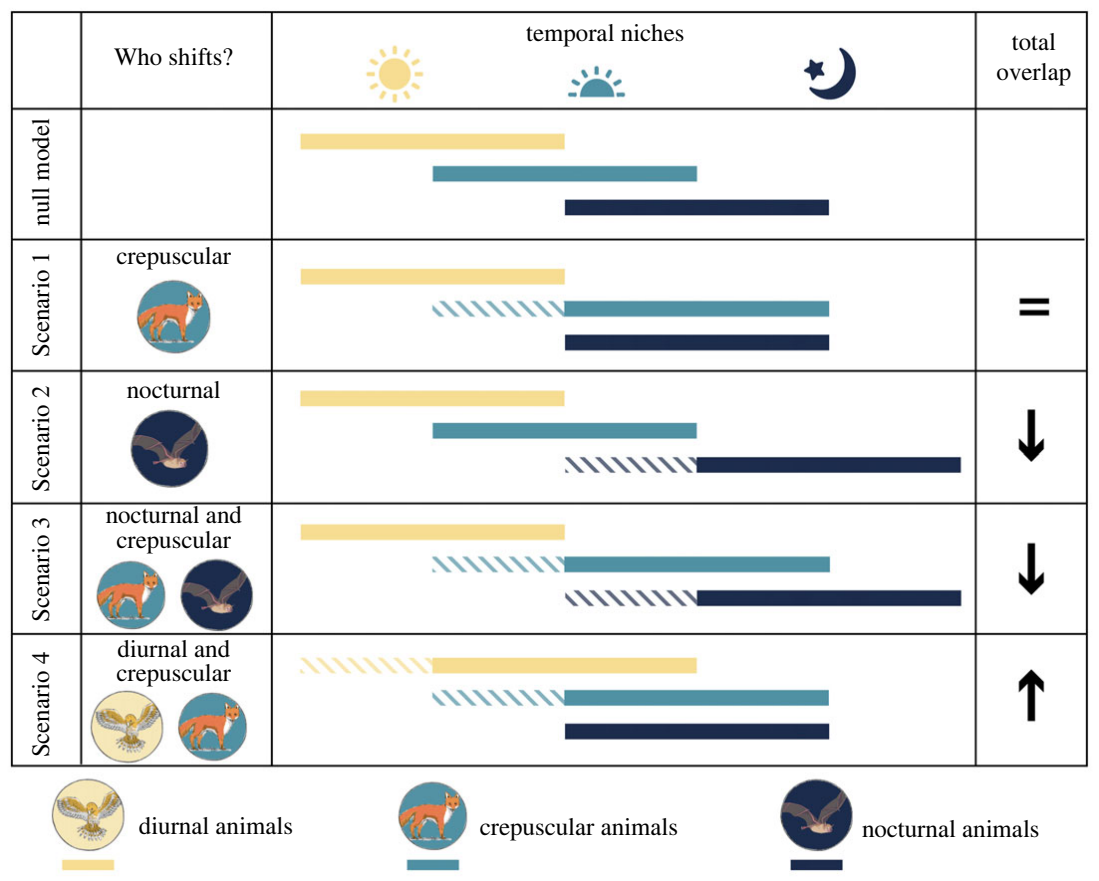
Concept of the food web modelling approach with the null model and four Scenarios (1–4). The effect of ALAN is modelled as a shift in the temporal niche of nocturnal, crepuscular and/or diurnal species, which affects their temporal niche overlap and therefore encounter probability. The change in total overlap indicates general niche separation or homogenization along the temporal dimension (Scenario 1: unaffected; Scenarios 2 and 3: niche separation and Scenario 4: niche homogenization). (Online version in colour.)

Using an ATN model (based on [72]), we generated food webs consisting of 20 plant (i.e. basal) and 40 animal species and simulated population dynamics in an ordinary differential equation system describing the rate of change in biomass densities for each species. The structure of food webs was determined by the body masses of organisms, which were randomly assigned (for more details regarding the model see the electronic supplementary material). In an additional step, we randomly assigned a temporal niche (diurnal, crepuscular or nocturnal) to all consumers and adjusted their interactions based on their temporal overlap. We assume that diurnal species and nocturnal species are not active at the same time and thus do not interact (i.e. their temporal overlap is 0). Crepuscular species overlap half of the time with diurnal species and half of the time with nocturnal species. Therefore, they overlap 50% of their activity time with diurnal and nocturnal species, respectively (figure 3, null model). The temporal overlap is used to scale the encounter probability between species from different temporal niches (see electronic supplementary material). For instance, as the temporal overlap of crepuscular species with diurnal and nocturnal species is of 0.5, their encounter rate is multiplied by 0.5, which directly affects the strength of their trophic interactions. As their temporal overlap is 0, nocturnal and diurnal species have an encounter probability of 0 and do not interact with each other. This created food webs where links between a diurnal predator and a nocturnal prey do not occur even if they would, based on body mass ratios.

### (b) Artificial light at night scenarios

We developed four scenarios of how animals might shift their temporal niches (i.e. related to the natural photoperiod) in response to ALAN without changing their total activity time (figure 3). Therefore, we simulated a gradient of how much the temporal niche of the different groups of diurnal, crepuscular and nocturnal species is shifted (hereafter ALAN effect). The ALAN effect takes values in [0, 0.5] to modify the temporal overlap defined in the null model. For example, an ALAN effect of 0.5 for crepuscular species would increase the niche overlap with nocturnal species by 0.5, leading to a full overlap (i.e. overlap of 1) of their temporal niches and simultaneously reduce their overlap with diurnal species from 0.5 to 0 (figure 3, Scenario 1). Thereby, ALAN will affect encounter probabilities and the interaction between species.

**Figure 4.**
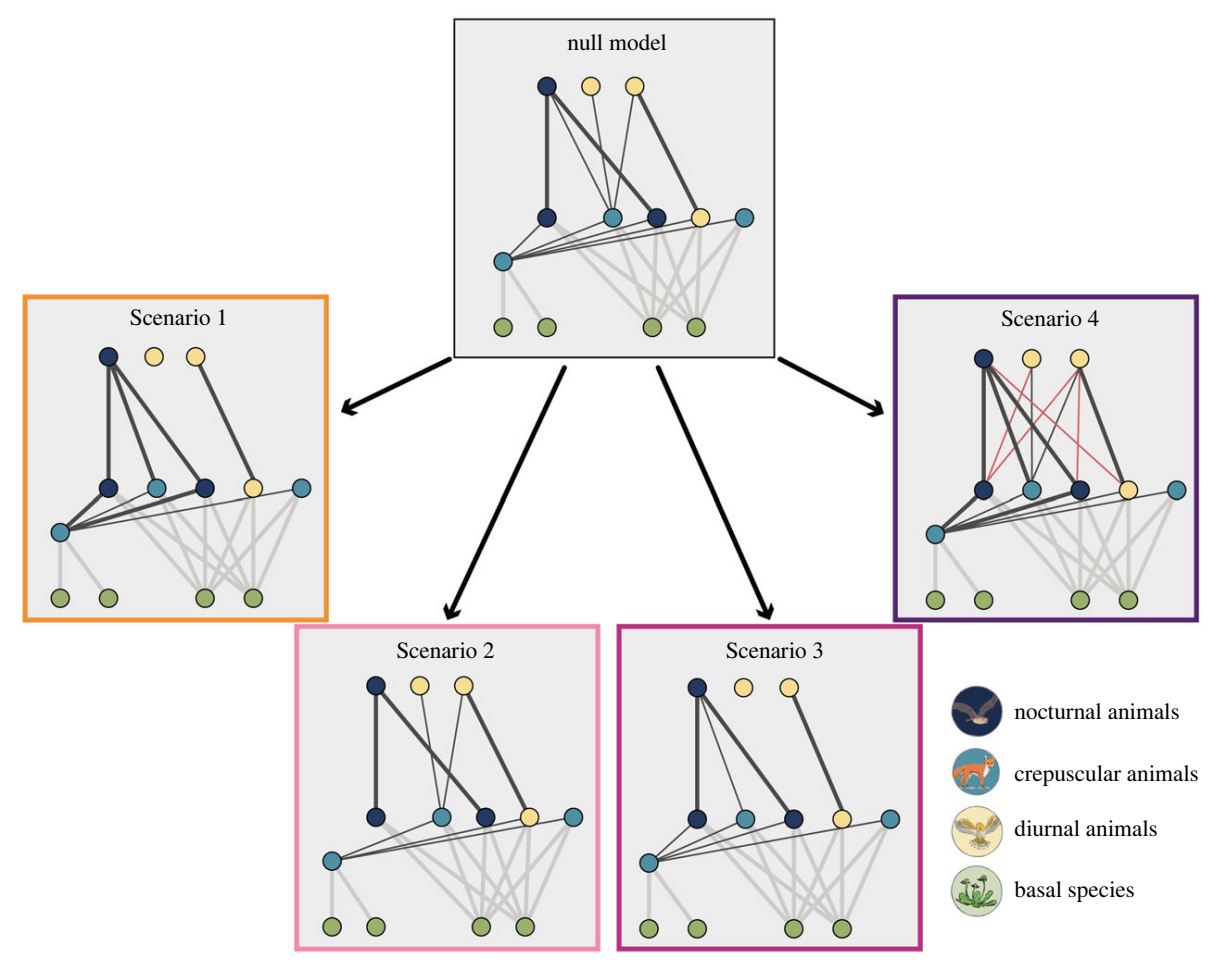
Illustration of how the different Scenarios of temporal shifts in response to ALAN can affect food web structure and interaction strengths. For illustration purposes, we depicted a food web of only 13 animal and four basal species (simulations were always done with 20 plant and 40 animal species) with the maximum shift in temporal niches of 0.5. Dashed red links indicate newly established links and the thickness of links represents the interaction strength. Dotted grey links represent links to basal species that are not directly affected by ALAN in the model. (Online version in colour.)

**In Scenario 1**, only crepuscular species react to ALAN by shifting their temporal niche towards the night. This shift results in an increased overlap and thus encounter probability of crepuscular species with nocturnal species and a reduced encounter probability with diurnal species. In this scenario, the total overlap of all species remains unaffected (figure 3, Scenario 1). This has implications for interaction strengths as well as the absence and presence of links in the food web. The higher encounter probability between crepuscular and nocturnal species would lead to stronger links between these species, while links between diurnal and crepuscular species become weaker or even disappear for the strongest value of the ALAN effect (figure 4, Scenario 1).

**In Scenario 2**, only nocturnal species react to ALAN by shifting their temporal niche further into the night, reducing their overlap with crepuscular species and restricting nocturnal predators to nocturnal prey. In this scenario, the total overlap among species is reduced (figure 3, Scenario 2). In the food web, this would lead to weaker or lost links between nocturnal and crepuscular species (figure 4, Scenario 2).

**In Scenario 3**, crepuscular as well as nocturnal species shift their temporal niche towards the night. This reduces the overlap of diurnal and crepuscular species but does not affect the overlap of crepuscular species with nocturnal ones. In this scenario, the total overlap among all species is also reduced (figure 3, Scenario 3). This leads to weaker or lost links between diurnal and crepuscular species in the food web, while links between nocturnal and crepuscular species are unaffected (figure 4, Scenario 3).

**In Scenario 4**, diurnal and crepuscular species shift their temporal niche into the night. While the overlap between crepuscular and diurnal species is unchanged, the overlap between crepuscular and nocturnal species increases. Moreover, this generates new overlap between diurnal and nocturnal species. Contrary to the previous scenarios, the total overlap among all species increases (figure 3, Scenario 4). In the food web, this means that links between crepuscular and nocturnal species become stronger and novel links between diurnal and nocturnal species are created (figure 4, Scenario 4).

### (c) Analyses

**Figure 5.**
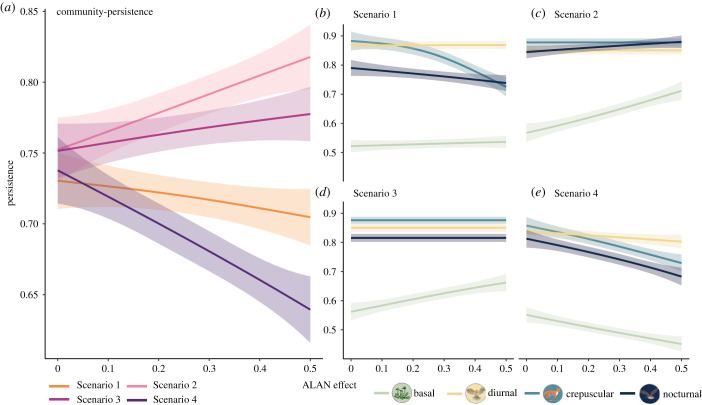
Impact of ALAN on the stability of modelled food webs. (*a*) Community persistence and (*b*–*e*) persistence of different groups (basal, crepuscular, nocturnal and diurnal species) in response to ALAN. The scenarios represent the different temporal niche shifts (figures 3 and 4). In **Scenario 1**, only crepuscular species shift (total niche overlap unaffected), in **Scenario 2**, only nocturnal species shift (total niche overlap decreased), in **Scenario 3**, nocturnal and crepuscular species shift (total niche overlap decreased) and in **Scenario 4**, diurnal and crepuscular species shift (total niche overlap increased). (Online version in colour.)

To assess how organisms within food webs respond to ALAN exposure, we recorded the persistence of the total community as the fraction of species not going extinct for each scenario as well as the persistence of basal, diurnal, crepuscular and nocturnal species separately. We consider a species extinct when its biomass density falls below a threshold of 10^−6^ g m^−2^ at any time during the simulation of the population dynamics. In figure 5, we plotted the relationships of persistence (*y*-axis) and the gradient of the temporal shift (ALAN effect on the *x*-axis) using the geom_smooth function (based on general additive models; method ‘GAM’) in ggplot2 in R.

### (d) Modelling results

The response of community persistence to ALAN was contingent on the scenario of temporal niche overlap, as depicted in figure 3. In Scenario 1, where the total niche overlap remained unchanged, ALAN had a slightly negative effect on overall persistence. Scenarios 2 and 3, in which the total niche overlap decreased, lead to slight positive effects. Conversely, when the total niche overlap increased in Scenario 4, community persistence decreased (figure 5*a*).

Irrespective of the overall response of the community, the persistence of individual groups (i.e. basal (plants), crepuscular, nocturnal, diurnal) exhibited a range of diverse and varying responses both within and across scenarios. The outcome depends on which of the groups shift their temporal niche in response to ALAN. For example, in Scenario 1, where crepuscular species shift their activity towards the night and therefore interact more with nocturnal species and less with diurnal species, basal and diurnal species generally showed neutral to slightly positive responses to ALAN. Crepuscular and nocturnal species, however, were more likely to go extinct due to their increased competition (figure 5*b*), which led to the overall slightly negative effect on community persistence (figure 5*a*). In Scenarios 2 and 3, only nocturnal or nocturnal and crepuscular species shift their niches. This leads to basal species showing an increase in persistence in response to ALAN while diurnal, crepuscular and nocturnal species were unaffected (figure 5*c*,*d*). This resulted in the slight positive overall effect observed at the community level (figure 5*a*). In Scenario 4, diurnal and crepuscular species shift their temporal niches towards the night and therefore overlap more with nocturnal species. This results in a higher extinction rate for basal, crepuscular and nocturnal species while diurnal species are less strongly affected (figure 5*e*). In this scenario, ALAN has a negative effect on community-level persistence leading to a substantial loss of diversity (figure 5*a*).

The pattern observed for basal species is potentially driven by the change in modularity of the food webs: the change of species overlap due to shifts in species' temporal niches leads to food webs where species from different groups are more or less isolated from each other (i.e. corresponding to food webs that are more or less modular). While in Scenario 1, modularity is least affected, it increases in Scenarios 2 & 3, and decreases in Scenario 4. An increase in modularity of the animal compartment is likely to reduce apparent competition among herbivores, allowing them better control of basal species [73], and to foster the coexistence of basal species [74]. Thus, our modelling approach—while explicitly modelling only one of the proposed pathways (i.e. shift in diel activity/functional roles)—also reveals a feedback effect to the pathway of primary producers.

Moreover, within communities, the response of individual animal groups (diurnal, crepuscular and nocturnal) relative to each other depends on their niche overlap and therefore the strength of their competition. This suggests that the impact of ALAN on overall community and basal species persistence is largely influenced by the degree and direction of changes in temporal niche overlap, rather than the specific group that responds to ALAN. However, the individual response of diurnal, crepuscular and nocturnal species to ALAN is heavily dependent on the specific temporal shifts that occur, i.e. what links in the food web are rewired (figure 4). However, our results focused on changes in species persistence, but more subtle effects such as changes in species dominance could also occur, calling for a more nuanced and comprehensive understanding of the ecological consequences of light pollution for food webs.

Overall, our modelling results suggest that by increasing or decreasing homogenization of diel niches along the temporal dimension, ALAN could act in a similar manner to spatial homogenization resulting from habitat loss or degradation, which has been shown to negatively impact biodiversity [75,76]. This stresses the need to document species' temporal shifts in response to ALAN at the community level and especially assess whether this leads to more overlapping temporal niches. Our model is a first formalization that can be extended to integrate various processes. First, we assumed that species keep their activity time constant under light pollution and only shift their temporal niche. However, organisms could also contract or expand their activity period in response to ALAN [77,78]. A natural extension would therefore be to relax our assumption and to assess how different biological responses to light pollution upscale to the community level. Because of our explicit formalization of time, the model also allows incorporation of the daily variations in environmental conditions such as temperature. Finally, we explore community responses to ALAN at a local scale, but more complex responses such as source-sink dynamics or rescue effects could arise from a patchy environment where some areas are experiencing ALAN and some are not. The meta-community extensions of the ATN framework [75,79] could offer a promising tool to tackle these questions.

## 3. Conclusion and outlook

Current empirical research on community-level and ecological-network responses to ALAN is still limited. To understand fully the impact of ALAN on ecological networks, we need to investigate how single-species responses to ALAN exposure are manifesting at the community level, which requires an understanding of the underlying mechanisms involved. To address this issue, we have conceptualized and reviewed four major pathways that can help uncover the mechanisms involved in affecting ecological networks. We demonstrated in a simplified worked example that theoretical modelling is a promising avenue to deepen our knowledge about the impact of ALAN on ecological networks and the underlying mechanisms. Such models can be expanded to include a wide range of factors, such as changes in temporal niche overlap (as shown here), overall increases or decreases in activity levels, and other ecological processes such as community assembly and meta-community dynamics. Additionally, interactive effects with other stressors, such as temperature, can be assessed. The insights gained from such an approach provide a more sophisticated set of hypotheses rooted in ecological theory, which can then be tested empirically to verify the underlying mechanisms. Overall, we present an integrative framework to help advance our predictive understanding of the community-level and ecological-network consequences of ALAN and their cascading effects on ecosystem functioning.

## Data Availability

The data are provided in the electronic supplementary material [80].
